# Nitrogen species specific phosphorus mineralization in temperate floodplain soils

**DOI:** 10.1038/s41598-021-96885-5

**Published:** 2021-08-31

**Authors:** Mary R. Arenberg, Yuji Arai

**Affiliations:** Department of Natural Resources and Environmental Sciences, University of Illinois at Urbana-Champaign, Urbana, IL 61801 USA

**Keywords:** Biogeochemistry, Environmental sciences

## Abstract

As an essential component of enzymes, higher N availability from agricultural runoff to forest soils may boost the activity of phosphatase, increasing the bioavailability of phosphate. The objective of this study was to evaluate P mineralization rates in temperate floodplain soils as a function of inorganic N species (i.e., ammonium and nitrate) and amendment rate (1.5–3.5 g N kg^−1^). Accordingly, the soil was amended with nitrate and ammonium, and P dynamics were monitored during a 40-day incubation. The addition of ammonium significantly boosted acid and alkaline phosphatase activity by 1.39 and 1.44 µmol *p*-nitrophenol P (pNP) g^−1^ h^−1^, respectively. The degree of increase was positively correlated with the amendment rate. Likewise, the P mineralization rate increased by 0.27 mg P kg^−1^ in the 3.5 g N kg^−1^ ammonium treatment. ^31^P nuclear magnetic resonance spectroscopic analysis further supported the reduction in organic orthophosphate diesters on day 30. Meanwhile, the addition of nitrate promoted P mineralization to a lesser degree but did not increase phosphatase activity. While floodplain soils have great potential to sequester anthropogenic P, high availability of inorganic N, especially ammonium, could promote P mineralization, potentially increasing P fertility and/or reducing P the sequestration capacity of floodplain soils.

## Introduction

As soils age and are depleted of the most easily accessible, inorganic phosphorus (P), the organic pool becomes a critical P sink and source for plants and microbes^1–3^. Mineralization regulates the transformation of organic P into bioavailable orthophosphate^4,5^. This process, however, is affected by various physicochemical parameters, including temperature, pH, moisture content, organic carbon (C), and nitrogen (N)^6–9^. Many soils have been heavily disturbed from their indigenous state through intensive applications of N^10^. Consequently, understanding the impacts of N deposition on P cycling is particularly important.

The N and P cycles are coupled through their biological demand but differ so substantially that either N or P may become limited compared to the other^11^. A frequently overlooked impact of N amendments is their potential to increase P fertility without the addition of P fertilizers. Nitrogen deposition allows organisms to invest N into the production of phosphatase^12^, of which N makes up 15–20%, by mass^13^. Thus, higher N availability may boost the activity of phosphatase, the enzyme that carries out the mineralization of organic P into bioavailable orthophosphate^14–16^. In a meta-analysis of 379 soils, total N was the strongest factor explaining spatial variation in phosphatase activity^12^. In our assessment of Illinois floodplain soils (n:120 surface soil samples), we observed strong correlations between soil N and (1) acid phosphatase activity (R^2^ = 0.48) and (2) alkaline phosphatase activity (R^2^ = 0.64)^17^.

Despite strong correlations between soil N and phosphatase activity, applying additional N does not necessarily increase phosphatase activity. In fact, N additions decreased phosphatase activity or had no significant effect in some soils^18–21^. However, N additions boosted phosphatase activity in a far greater number of soils, including in agricultural, forest, and grassland systems^16,22–29^. Based on these contradicting responses, it was hypothesized that the form of N added and/or its amendment rate could have resulted in variable phosphatase response. In a forest soil, for example, only inorganic and organic N applied together boosted phosphatase^30^. Most studies added N as ammonium nitrate (NH_4_NO_3_)^22,23,25,26,31^, and it is not clear which N species impacted the results. The N fertilizer amendment rate and/or the amount of N deposition also varied in studies reviewed in this section^22,28,31–33^. Changes in phosphatase activity were typically but not universally correlated with the rate of N application.

While the effects of N on phosphatase activity have been extensively studied, the broader impacts on biological P cycling (e.g., rate of P mineralization, organic P concentration, and P speciation) have been seldomly investigated. It has been hypothesized that N deposition may delay the onset of P limitation as soils age^32,34^. Through the reduction of P solubilizing bacteria and the increase of phosphatase production, in certain soils, these N additions may even change the dominant P processes from solubilization of inorganic P to mineralization of organic P^24^. Increased phosphatase activity could subsequently induce greater mineralization of organic P into labile inorganic P, which is much more susceptible to loss through leaching^35^. In the few studies where these broader P dynamics were examined, mixed results were reported. In northeastern US hardwood forests, N boosted phosphatase activity but did not, in turn, increase resin extractable-P that is an indicator of bioavailable P^36^. In a forest in Northeastern China, certain N treatments increased alkaline phosphatase activity while also increasing microbial biomass carbon, orthophosphate, and orthophosphate monoesters and diesters^30^. In deciduous forest soils in Northeastern U.S., long-term N deposition increased P mineralization rates while decreasing total P in the organic horizon^37^. These contradicting results motivate further investigation of the effects of N deposition on P mineralization dynamics.

The objective of this study was to evaluate P mineralization rates as a function of (1) inorganic N species (i.e., ammonium as NH_4_Cl and nitrate as NaNO_3_) and (2) N amendment rate. Laboratory incubation experiments were carried out using temperate forest floodplain soils from the Midwestern United States. The soil was held under a constant moisture content, and its P dynamics were examined using wet chemistry analysis and solution ^31^P Nuclear Magnetic Resonance spectroscopy (NMR). In addition to acid and alkaline phosphatase, important biochemical parameters (e.g., labile inorganic and total organic P, orthophosphate monoesters, and orthophosphate diesters) related to P mineralization were monitored.

## Results and discussion

### Soil characterization

Before the incubation, various physicochemical parameters of the soil were characterized. The initial soil pH_water_ was 6.24 ± 0.04. The total base saturation was 98.51% while the total cation exchange capacity was 34.57 meq/100 g. The soil contained 8.32% organic matter and 31 ± 13 g organic C kg^−1^. The total and organic P of the soil were 360 ± 50 mg kg^−1^ and 161 ± 55 mg kg^−1^, respectively.

### Phosphatase activity

**Figure 1:**
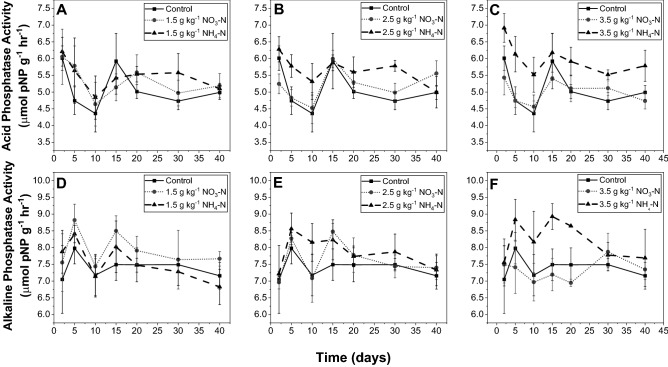
Acid phosphatase activity (**A**–**C**) and alkaline phosphatase activity (**D**–**F**) during a 40-day incubation as a function of nitrate or ammonium amendment 1.5–3.5 mg N kg^−1^ The error bars indicate the standard deviation.

The acid and alkaline phosphomonoesterase activities of the soils were analyzed as a function of inorganic N amendment species, N amendment rate, and time (Fig. 1). These two enzymes are optimized under varying pH conditions and originate from different sources^38,39^. While acid phosphatases are primarily released by plants and fungi^40^, alkaline phosphatases are produced by soil microbes, namely bacteria^41^. In a soil with a pH of 6.24, both enzymes are relevant to P mineralization. It is important to note that the traditional assays conducted in this present study measure the potential enzyme activity, rather than the actual activity^42^.

Ammonium significantly boosted the acid phosphatase activity relative to the control on days 5, 10, 20, and 20 and to the nitrate treatment on days 2, 5, 10, and 30 (Fig. 1A; Table A1). The acid phosphatase activity in the control and nitrate treatment were not significantly different from each other during any sampling period (Table A1). Meanwhile, amendment rate had a less pronounced effect on acid phosphatase activity while considering both nitrate and ammonium (Fig. 1B; Table A2). However, analyzing nitrate and ammonium separately revealed that the effect of amendment rate depended on the inorganic N species. Linear fitting revealed very weak relationships between nitrate application rate and change in acid phosphatase activity (Fig. 1C; Table A3). However, ammonium amendment rate was positively correlated with acid phosphatase activity. These positive correlations were statistically significant on days 5, 10, and 20 (Table A3).

Moreover, alkaline phosphatase activity was significantly boosted by ammonium on days 15 and 20 relative to the control and on day 20 relative to nitrate (Fig. 1D–F; Table A4). Likewise, the effect of amendment rate varied between the nitrate and ammonium (Tables A5, A6). Correlations between nitrate addition and alkaline phosphatase activity were weak and not significant. Conversely, the amount of ammonium added was positively correlated with alkaline phosphatase activity for nearly all sampling periods and significantly correlated on days 5, 15, and 20.

Ammonium amendments increased acid and alkaline phosphatase activities to a degree dependent on the rate of application. This finding is consistent with some previous studies, in which researchers also observed ammonium-induced increases in the activity of acid and/or alkaline phosphatases^33,43,44^. However, other studies concluded no effect^18^ or a dampening of phosphatase activity caused by ammonium^19,45^. The additional ammonium likely enabled microbes to invest more N into the production of phosphatase. Meanwhile, nitrate did not have a significant effect on acid or alkaline phosphatase activity, which is in accordance with previous work^21,44,46^. This discrepancy is likely a result of the preferred N form by microbes. Nitrate assimilation consumes large amounts of energy^47,48^. Thus, ammonium is generally the preferred N form by heterotrophic bacteria that produce phosphatase enzymes^49^.

### Labile inorganic P and rates of P mineralization

Bicarbonate extractable, labile inorganic P represents bioavailable P in the soil solution and sorbed onto calcium carbonates and Fe oxides^50^. While it does not directly measure gross P mineralization, tracking its changes is a useful estimate of P mineralization among different treatments within the same soil series. Furthermore, it is reflective of plant-available P^51^.

**Figure 2:**
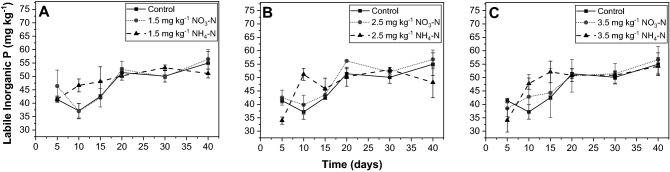
Labile inorganic P over the course of 40-day incubation, as a function of N species and amendment rate: (**A**) 1.5 mg kg^−1^, (**B**) 2.5 mg kg^−1^, and (**C**) 3.5 mg kg^−1^. All analyses were conducted in triplicate. The error bars indicate the standard deviation.

Throughout the incubation, labile inorganic P increased across all treatments, to differing degrees (Fig. 2). In the control, labile inorganic P varied from 37.17 to 54.98 mg kg^−1^ while it ranged from 37.06 to 56.43 mg kg^−1^ in the nitrate 1 treatment and 46.64–53.20 mg kg^−1^ in the ammonium 1 treatment (Fig. 2A). Furthermore, labile inorganic P increased 39.85–56.75 mg kg^−1^ in the nitrate 2 treatment and 45.86–52.98 mg kg^−1^ in the ammonium 2 treatment (Fig. 2B). Labile inorganic P varied from 42.85 to 56.65 mg kg^−1^ in the nitrate 3 treatment and 47.75–54.20 mg kg^−1^ in the ammonium 3 treatment (Fig. 2C). These values are in accordance with those reported for a French riparian forest^52^ and a New Hampshire hardwood forest soil^53^. At a 0.05 level, labile inorganic P was significantly greater in the ammonium treatments relative to the control on day 10 and relative to the nitrate treatments on days 5 and 10 (Table A7).

To calculate the rate of P mineralization, a zero-order kinetic model was used, in which changes in labile inorganic P were plotted against time. The rates, measured in mg P kg^−1^ day^−1^, were calculated over two different time periods: days 0–20 and days 20–40. These periods were separated because changes in labile inorganic P tended to slow greatly after day 20. Increases in labile inorganic P represent net P mineralization while decreases in labile inorganic P signify net immobilization^54,55^. Using labile P to calculate P mineralization may overlook mineralized P that quickly sorbs onto mineral surfaces or is up-taken by microbes^51,56,57^. Nevertheless, NaHCO_3_ measures bioavailable P in the soil solution and adsorbed onto calcium carbonates and Fe oxides^50^. Thus, it is useful for comparing net mineralization between different treatments within the same soil series, especially when also considering an unamended control^56^. Evaluation of mineralization dynamics is further supported by ^31^P NMR speciation and assessment of organic P.

Statistical assessment of labile inorganic P of N amendment rate is summarized in Table A8. From days 0–20, P mineralization rates varied throughout the treatments as follows: ammonium 3 >> ammonium 1 ~ nitrate 2 > ammonium 2 >> nitrate 3 > nitrate 1 >>  control (Table 1). During this period, all rates were positive, signifying P mineralization across all treatments. Nevertheless, there was a wide variation among the treatments. The highest rate occurred in the ammonium 3 treatment at 0.85 mg P kg^−1^ day^−1^ (R^2^ = 0.89) while the lowest rate was in the control at 0.58 mg P kg^−1^ day^−1^ (R^2^ = 0.84). Meanwhile, P mineralization rates significantly dropped from days 20–40, and these rates varied as follows: nitrate 3 >  > nitrate 1 ~ control ~ ammonium 3 >> ammonium 1 ~ nitrate 2   >> ammonium 2. During this period, mineralization occurred most quickly in nitrate 3 at a rate of 0.30 mg P kg^−1^ day^−1^ (R^2^ = 0.85) and most slowly in ammonium 2 at a rate of – 0.11 mg P kg^−1^ day^−1^ (R^2^ = 0.19). These mineralization rates fall within the range of those previously reported for forest and floodplain soils^4,58–61^.

**Table 1:** Phosphorus mineralization rates from days 0–15 and days 15–40, as affected by N species and amendment rate.

Treatment	Amendment rate (mg N kg^−1^)	Days 0–20		Days 20–40	
		k (mg P kg^−1^ day^−1^)	R^2^	k (mg P kg^−1^ day^−1^)	R^2^
Control	N/A	0.58	0.84	0.18	0.49
Nitrate	1.5	0.63	0.74	0.19	0.33
	2.5	0.77	0.88	0.03	0.01
	3.5	0.65	0.88	0.30	0.85
Ammonium	1.5	0.78	0.98	0.04	0.08
	2.5	0.75	0.82	− 0.11	0.19
	3.5	0.85	0.89	0.17	0.75

The rates were calculated using a zero-order kinetic model, where P _min_ = k · time. P_min_ equals changes in labile inorganic P (mg P kg^−1^ day^−1^).

During days 0–20, the highest rate of mineralization occurred in ammonium 3, which corresponds to the highest acid and alkaline phosphatase activity. Thus, ammonium-induced increases in phosphatase activity, in turn, led to significant increases in P mineralization. A previous study observed positive correlations between alkaline phosphatase and labile inorganic P but did not specifically estimate P mineralization rates^46^. Phosphorus reaction dynamics slowed down following day 20 likely because the most easily accessible organic P within the soil had already been mineralized. Other researchers observed similar stabilizations in labile/extractable P at approximately days 10–20 of their laboratory incubations^55,59^. Additionally, changes in phosphatase activity tended to slow down around this same period.

### Soil pH and organic P

**Figure 3:**
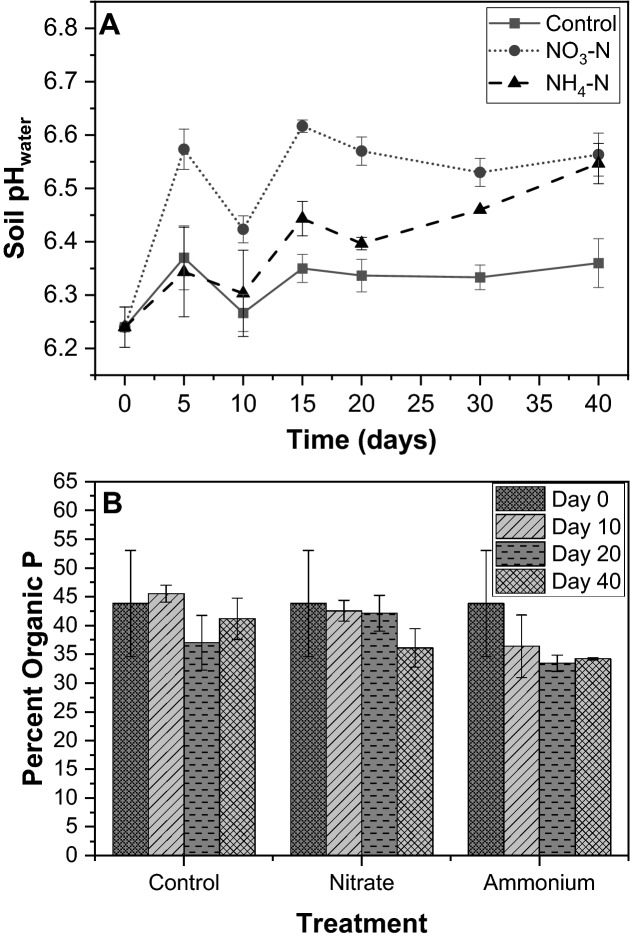
(**A**) Soil pH_water_ over the course of 40-day incubation in the control and nitrate, and ammonium treatments (3.5 mg N kg^−1^). All analyses were conducted in triplicate. The error bars indicate the standard deviation. (**B**) Percent organic P on days 0, 10, 20, and 40 in the control and nitrate and ammonium treatments (3.5 mg N kg^−1^). All analyses were conducted in triplicate. The error bars indicate the standard deviation.

Throughout the incubation, soil pH _water_ was monitored to track the effect of the nitrate and ammonium amendments (Fig. 3A). Both inorganic N species led to an increase in pH, to differing degrees. The pH of the control increased to 6.37 ± 0.06 on day 5 and then remained steady and lower than the N-amended treatments for the duration of the incubation. The nitrate caused a large increase in the soil pH from day 0 to 5, on which it was 6.57 ± 0.04 in the nitrate 3 treatment. This large increase is likely due to nitrate assimilation, which is an alkalinizing process that consumes H^+^^62,63^. With some slight fluctuation, the pH in nitrate 3 remained steady and was 6.56 ± 0.04 on day 40. The pH of ammonium 3, meanwhile, gradually increased throughout the incubation, from 6.34 on day 5 to 6.55 on day 40. pKa of ammonium (9.23) seemed to contribute to a slight pH increase. There was no sign of soil acidification by N input..

Total organic P, normalized as percent of total P, was measured on days 0, 10, 20, and 40 (Fig. 3B, Table A9). Initially, the soil contained 44 ± 9% organic P. In the control, organic P fluctuated during the incubation to 46 ± 1%, 37 ± 5%, and 41 ± 4% on days 10, 20, and 40, respectively. The inorganic N amendments led to decreases in percent organic P in both nitrate 3 and ammonium 3. On one hand, in nitrate 3, this dropped in percent organic P mostly occurred between days 20 and 40, on which it comprised 36 ± 3% of total P. On the other hand, in ammonium 3, organic P decreased mostly between days 0 and 10, on which it made up 36 ± 5% of total P. On day 40, organic P made up 34.2 ± 0.2% of total P in ammonium 3. At a significance level of 0.05, percent organic P was significantly less in ammonium 3 than the control on days 10 and 40 and nitrate 3 on day 20 (Table A9). Linear fitting revealed a significant, negative correlation between organic P and labile inorganic P (Pearson’s r =  − 0.699), demonstrating that P availability increased as organic P decreased. These reductions in organic P throughout the incubation are further evidence that increased P mineralization occurred in the ammonium 3 and nitrate 3 treatments^64^.

### Inorganic and organic ^31^P NMR speciation

**Figure 4:**
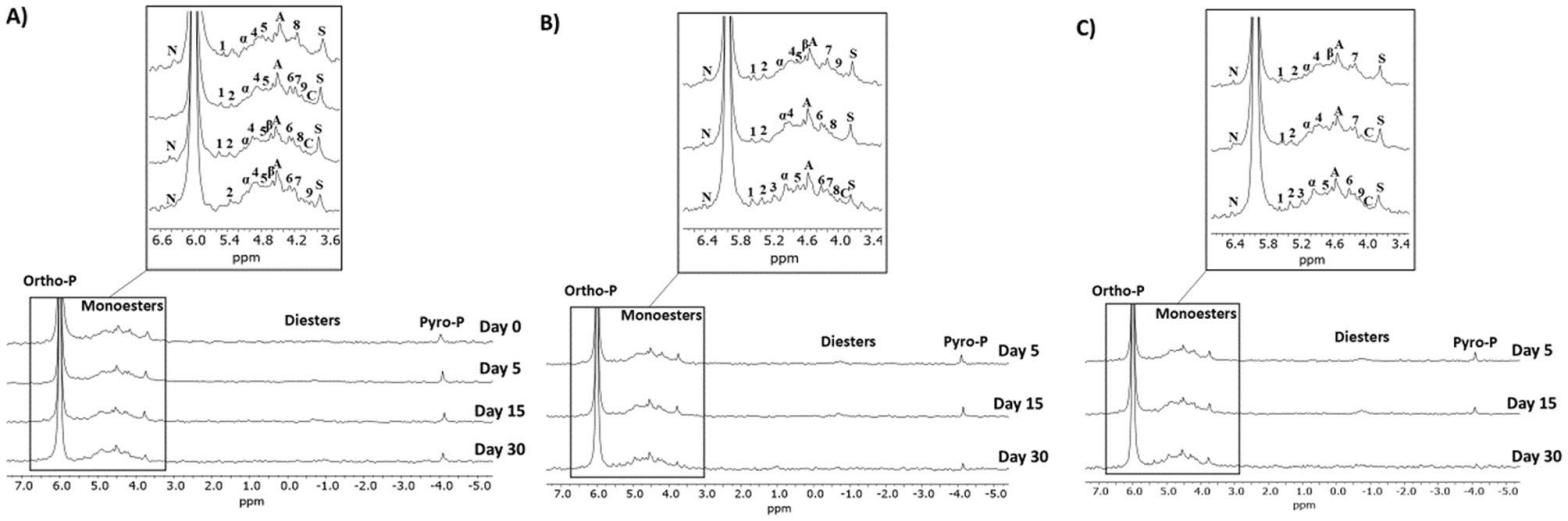
^31^P solution-state NMR spectra of soil extracts from the (**A**) control, (**B**) nitrate (3.5 mg N kg^−1^), and (**C**) ammonium (3.5 mg N kg^−1^), on days 5, 15, and 30 of the incubation as well as day 0 of the A) control. Soils were extracted with NaOH-EDTA. Peaks assigned as Ortho-P: orthophosphate, N: neo-IHP, α: α-glycerophosphate, β: β-glycerophosphate, A: adenosine 5′ monophosphate, C: choline phosphate, S: scyllo-IHP, and Pyro-P: pyrophosphate. Unknown monoester peaks are denoted by 1, 2, 3, 4, 5, 6, 7, 8, and 9. The region from ~ 5.8–3.4 ppm includes orthophosphate monoesters as well as degradation products of orthophosphate diesters, which are also found in the − 0.25 to − 0.75 ppm region.

Temporal changes in the P speciation of the soil, as affected by inorganic N amendment species, were monitored using ^31^P solution-state NMR spectroscopy (Fig. 4). The objectives of using NMR spectroscopy in this experiment were (1) to further monitor changes in inorganic P (i.e., orthophosphate and pyrophosphate) relative to organic P (i.e., orthophosphate monoesters and diesters) (Table 2) and (2) identify specific P compounds and observe their evolution over time. It is important to note that the percent organic P determined by wet chemistry and ^31^P NMR were not in accordance. This discrepancy arises from the percent recovery rate of total P in the NaOH-EDTA soil extracts, which was determined to be 63 ± 7%. Chemical shifts of peaks of various P compounds are summarized in Table A10.

**Table 2:** Percent of inorganic P, organic P, inorganic orthophosphate, inorganic pyrophosphate, orthophosphate monoesters, and orthophosphate diesters, as determined by ^31^P NMR.

Treatment	Time (days)	Inorganic P	Organic P	Ortho-P	Pyro-P	P-mono	P-diest
Control	0	76.1	23.9	74.1	2.1	22.1	1.8
	5	77.0	23.0	74.5	2.5	20.5	2.5
	15	77.4	22.6	75.0	2.4	17.7	3.8
	30	78.2	21.8	76.3	1.9	15.6	6.1
Nitrate	5	81.4	18.6	79.5	1.9	14.2	4.4
	15	81.0	19.0	79.0	0.2	14.3	4.7
	30	81.5	18.5	80.3	1.2	16.9	1.7
Ammonium	5	79.5	20.5	77.4	2.1	14.3	6.2
	15	81.4	18.6	80.0	1.4	13.9	4.7
	30	80.1	19.9	79.4	0.7	17.6	2.3

On days 0, 5, 15, and 40, NMR spectra were collected for the control and the treatments to which the highest rates (3.5 g N kg^−1^) of nitrate and ammonium were applied. Day 0 was chosen to assess the initial P speciation of the soil. Wet chemistry analysis suggested large changes in labile inorganic P and phosphatase activity during the first 15 days of the incubation. Thus, days 5 and 15 were chosen to monitor how these changes affected P speciation. Phosphorus reactions markedly slowed down in the latter half of the incubation, so changes in P speciation were estimated again on day 30.

Initially, the soil contained 76.1% inorganic P and 23.9% organic P. In the control, which did not receive any amendments, P speciation changed slightly over time. Inorganic P, including orthophosphate and pyrophosphate, comprised 77.0%, 77.4%, and 78.2% of total P on days 5, 15, and 30, respectively while organic P, including orthophosphate monoesters and diesters, made up 23.0%, 21.5%, and 21.8% over the same period. Overall, inorganic P increased in the control by just 2.8% over the first 30 days while organic P decreased by the same degree, signifying a small degree of P mineralization.

On day 5, organic P was lower in the nitrate and ammonium treatments than the control while inorganic P was higher than the control. This same trend persisted on days 15 and 30 as well. On days 5, 15, and 30, organic P was 3.3–4.4% lower in the nitrate treatment and 1.9–4.0% lower in the ammonium treatment. These relative proportions of organic and inorganic P further support the observation that the application of inorganic N amendments increased soil P mineralization. Orthophosphate diesters initially had increased in the nitrate and ammonium treatments compared to the initial soil. Because orthophosphate diesters reflect microbial activity^65^, the initial N additions may have boosted microbial biomass and activity (e.g., the production of phosphatases). The diesters then consistently decreased from days 5–30. As greater phosphatase activity increased P mineralization, those diesters were likely mineralized. By contrast, monoesters are much less susceptible to degradation due to their strong adsorption onto soil minerals^66–68^.

Various species of orthophosphate monoesters and diesters were identified in the spectra. Compounds detected through spiking experiments include α-Glycerophosphate and β-glycerophosphate, which are diester degradation products, as well as monoesters: adenosine 5′ monophosphate and choline phosphate (Figure A1). Neo- and scyllo-IHP were clearly identified through the consultation of past literature because they are not commercially available for spiking^69–71^. Nine monoester peaks (chemical shifts: 5.53 ± 0.03, 5.36 ± 0.02, 5.15 ± 0.04, 4.91 ± 0.03, 4.74 ± 0.02, 4.30 ± 0.01, 4.21 ± 0.02, 4.14 ± 0.02, and 4.08 ± 0.01) could not be specifically identified. The latter four peaks likely encompass various mononucleotides, but the precise species would need to be confirmed through additional spiking experiments^72^. The monoester compounds, both those identified and unidentified, most likely include recalcitrant organic matter while the diesters include new compound synthesized by microbes during immobilization^73^.

While phytic acid tends to be the dominant organic P species in soil due to its strong affinity for soil minerals, on which it adsorbs and is protected from degradation^74,75^, it, surprisingly, was not specifically identified in the spectra of the present study. A spiking experiment with phytic acid showed where the four peaks would be found (Figure A1); they may have been present but convoluted by larger peaks (i.e., α-glycerophosphate and adenosine 5′ monophosphate). However, other inositol hexaphosphates (i.e., neo- and scyllo-IHP), which are similarly resistant to degradation, were detected in the spectra.

### Effect of nitrogen species on phosphatase and P mineralization parameters

Inorganic N species type significantly affected phosphatase activity and, subsequently, P mineralization dynamics in this Illinois floodplain soil. Due to lower energy costs, heterotrophic bacteria, which produce phosphatase enzymes, prefer to uptake ammonium over nitrate^48,49^. This preference likely caused ammonium to significantly boost acid and alkaline phosphatase activity in the present study. In turn, the higher phosphatase activity in the ammonium treatments significantly increased labile inorganic P and P mineralization rates during the first 20 days of the incubation. This elevated P mineralization was further observed in the decreases in percent organic P and organic P speciation. Ammonium stayed relatively constant in the 2.5 g kg^−1^ and 3.5 g kg^−1^ treatments while nitrate levels remained below the control, signifying minimal nitrification (Figure A2).

Meanwhile nitrate did not increase phosphatase activity. Still, mineralization occurred in the nitrate treatment, particularly in nitrate 3. While P mineralization rate was lower than ammonium 3 during days 0–20, percent organic P in nitrate 3 was not significantly different than ammonium 3 by day 40. This was further supported by P speciation, where organic P, especially orthophosphate diesters, decreased to the same degree in nitrate 3 as in ammonium 3. Thus, both ammonium and nitrate induced P mineralization.

Most previous studies either applied these two N species together or one of the two by itself. Additionally, past researchers tended to concentrate on the effects of inorganic N on phosphatase activity, rather than broader P mineralization dynamics. However, in one study, divergent effects of nitrate and ammonium on C mineralization were observed^76^. Both inorganic N species, but especially ammonium, enhanced C mineralization. The reviewers speculate that this divergence resulted from the differing interactions of the two N species with the soil matrix. While positively charged ammonium readily adsorbs onto soil particles, negatively charged nitrate is much more susceptible to loss through leaching^76^. Moreover, increasing atmospheric CO_2_ is inhibitory to nitrate assimilation. Thus, researchers predict that ammonium uptake will become more and more favored as climate change progresses^77^. This trend could potentially accelerate ammonium-induced P mineralization.

## Conclusions

Inorganic N additions tended to induce greater phosphatase activity and consequently accelerated P mineralization in an Illinois floodplain during the first 20 days of a laboratory incubation. However, the form and amount of N applied matter. Ammonium, which is preferred by heterotrophic microbes had a stronger impact and significantly boosted acid and alkaline phosphatase activity to a degree dependent upon application rate. In turn, ammonium also increased P mineralization rates and decreased organic P and orthophosphate diesters. While nitrate did not significantly affect phosphatase activity, it did induce mineralization to a lesser degree than ammonium. This discrepancy between the two N sources likely results from microbial preference for ammonium. In agricultural runoff, nitrate is predominant N species. One should not expect nitrate to largely impact the P sequestration in the floodplain soils. If excessive N deposition in Midwestern floodplain soils reduces their P sequestration capacity, nitrate needs to be reduced to ammonium. Redox chemistry of soils plays an important role in N specific P mineralization in floodplain soils. Finally, the concentration threshold at which N induces P mineralization should be further investigated in assessing P fertility of soils.

## Materials and methods

### Materials

In July 2019, surface soil (0–25 cm deep, 25 cm wide) from the Ap horizon was collected from the floodplain of Robert Allerton Park in Monticello, Illinois (N 39° 59.766′ W 88° 38.914′). The bottomland forest of Robert Allerton Park is flooded approximately 10% of the time, most commonly in March and April^78^. Over an 8-year period, sedimentation ranged from 0.6 to 5.6 cm^79^. Sediment depth, mass, or particle size does not significantly correlate with distance from the river channel or with elevation^79^. Total C, N, P, S, and base cations were greater in the bottomland site than its surrounding upland due to floodwater sedimentation^17,79^. The floodplain is dominated by silver maple (*Acer saccharinum* L.)^79^. The soil is very poorly drained Sawmill silty clay loam (fine-silty, mixed, superactive, mesic Cumulic Endoaquolls). The initial total N, nitrate, and ammonium in the soil were 1.44 g kg^−1^, 30.95 mg kg^−1^, and 14.16 mg kg^−1^, respectively.

Soil physicochemical properties, including soil pH_water_^80^, total base saturation^81^, total exchange capacity^82^, organic matter^83^, organic C^84^, and total and organic P^85^ were characterized. The total P and organic P concentrations were determined colorimetrically, using the molybdate blue method^86^ and the modified Asher molybdate blue method^87^, respectively. All chemicals used in this study were of the American Chemical Society (ACS) grade. Ultrapure water (18.2 MΩ cm) was used for experiments.

### Incubation study

Within five days of collection and following storage at ambient room temperature (~ 23 °C), sodium nitrate and ammonium chloride were applied to the soil at three different rates to represent different levels of N deposition. The rates applied include (1) 1.5 g N kg^−1^, (2) 2.5 g N kg^−1^, and (3) 3.5 g N kg^−1^. Henceforth, these application rates will be referred to as nitrate/ammonium 1, nitrate/ammonium 2, and nitrate/ammonium 3. The rates were selected following an evaluation of 20 sampling sites throughout the floodplain of Allerton Park, where total N ranged from 1.44 to 3.85 g kg^−1^^17^. The initial total N of the selected soil was at the bottom of this range (i.e., 1.44 g kg^−1^). The first N rate (i.e., 1.5 g N kg^−1^) brought the total N to the middle of the range while the second N rate (i.e., 2.5 g N kg^−1^) boosted soil N to the top of the range. Finally, the highest N rate (i.e., 3.5 N g kg^−1^) increased the soil N concentration to 1 g N kg^−1^ beyond the range. The N amendments were applied as solutions to adjust the gravimetric soil moisture to 0.46 g water g^−1^ soil. Because of high plasticity of clayey soil, the soils and N solutions were homogenized through thorough hand mixing for 30 min.

Triplicates of each treatment and the control were prepared. Immediately following application of nitrate and ammonium, the samples were incubated for 40 d at ambient room temperature (~ 23 °C) and constant moisture. Throughout the incubation, kinetic subsamples were removed on days 2, 5, 10, 15, 20, 30, and 40. The kinetic samples weighed 15 g on day 2 and 30 g on all the other sampling days. Thus approximately 195 g, or 43% of each total sample was removed through sampling by the end of the experiment. The subsamples were analyzed to assess soil pH_water_^80^, phosphatase activity^88^, and P mineralization- immobilization dynamics: labile inorganic P^89^ and organic P^85^. The P speciation of 0, 5, 15 and 30 day samples was carried out using ^31^P solution-state NMR spectroscopy^90,91^.

### Chemical analyses

First labile inorganic P, which includes inorganic P that is weakly adsorbed onto various soil surfaces, including carbonates, sesquioxides, or crystalline minerals^92^, was analyzed. NaHCO_3_ is suitable to assess labile inorganic P in this soil because it extracts dissolved P as well as P adsorbed on carbonate and Fe- and Al-oxide surfaces for soils > pH 6.0^50,93^. Labile inorganic P was measured after 2.5 g soil was extracted with 20 mL of 0.5 M NaHCO_3_, pH 8.5^89^. The P concentration of the extracts were determined colorimetrically at 888 nm, using the modified Asher molybdate blue method^87^.

Organic P was analyzed using a modified method of Bowman^85^. The moist soil equivalent of 1.0 g of dry soil was sequentially extracted, first with 1.5 mL 18 M H_2_SO_4_, subsequently diluted to 25 mL, and then with 49 mL 0.5 M NaOH. After adjusting the pH of the extracts to 5.4, orthophosphate concentrations were determined using the modified Asher molybdate blue method^87^. Inorganic P concentrations equaled the sum of the orthophosphate concentrations of strong acid and dilute base extracts. Total P was quantified colorimetrically at 882 nm, using a modified molybdate blue method^86^, following the separate digestions of 2-mL and 4-mL aliquots of the acid and base extracts, respectively, with 2 mL 5.5 M H_2_SO_4_ (Fisher Scientific International, Inc., Fair Lawn, NJ, USA) and 0.5 g K_2_S_2_O_8_ (Sigma-Aldrich, St. Louis, Missouri, USA) for 30 min at 150 °C^85^. The total P concentration for each sample was calculated as the sum of the P concentrations of the sample’s acid and base extracts while the organic P concentration of each sample was estimated as the difference between the total P and inorganic P concentrations.

Acid phosphatases are most commonly released by plant roots and fungi^94–96^ while alkaline phosphatases are primarily excreted by bacteria^40,97^. The acid and alkaline phosphomonoesterase activities of the soil were measured as indirect evidence of P mineralization through p-nitrophenyl phosphate assays of moist soil at pH 6.5 and 11, respectively^88^. These traditional assays measure the potential enzyme activity, rather than the actual activity^42^. Controls for each soil sample were also prepared to account for the color not derived from p-nitrophenol released through phosphatase activity. Following the assay, the p-nitrophenol (pNP) concentrations of the filtrates were determined colorimetrically. Phosphatase activity was then estimated using the formula below:

$$\text{Phosphatase} \; \text {activity} ({\upmu \text{mol} \; \text{pNP}}/\text{g}\; \text{soil} \cdot \text{hour})=\frac{Treatment \; \mu {{mol}} \; P\text{-}nitrophenol - Control \; \mu {{mol}} \; P\text{-}nitrophenol}{g \; soil \cdot hour} \; $$

To measure NO_3_-N & NH_4_-N, the moist soil was combined with 2 M KCl (Fisher Scientific International, Inc., Fair Lawn, NJ, USA) and shaken for 2 h at 150 rpm^98^. The extracts were auto-analyzed to assess NH_4_-N while NO_3_-N was measured colorimetrically at 220 nm^99^.

### ^31^P nuclear magnetic resonance spectroscopy

To understand P speciation in soils, ^31^P solution-state NMR analysis was conducted on NaOH-ethylenediaminetetraacetic acid (EDTA) extracted soil samples to evaluate treatment effect on speciation of organic P throughout the incubation and^90,91,100–102^. First, 1.5 g of soil/plant residue was mixed with 30 mL of 0.25 M NaOH and 0.05 M Na_2_EDTA and shaken for 16 h^90,91^. Extracts were centrifuged for 20 min at 2862 g, and the supernatants were then frozen and lyophilized. Then, 0.6 g of lyophilized extract was dissolved in 0.2 mL of D_2_O and 1.8 mL of 1.0 M NaOH and 0.1 M Na_2_EDTA and centrifuged for 20 min at 2516 g To collect ^31^P-NMR spectra, a Varian Unity Inova 600 MHz spectrometer with a 5 mm Varian AutoTuneX 1H/X PFG Z probe, X = ^31^P-^15^N (− 80 to + 130 °C), running at a frequency of 242.95 MHz and a pulse width of 90°, was utilized. The NMR parameters included 0.69 s acquisition time and 6.6 s relaxation delay. Extracts were analyzed with a D_2_O field lock at 25 °C. The longitudinal relaxation (T1) time was specifically determined for each sample. Approximately 2000 scans were collected for each sample. The total experiment time was approximately 4 h per sample. Because of costs, only one replicate of each sample was analyzed.

All NMR spectra processing was completed using Mestrenova software (Mestrelab Research, S.L., Santiago de Compostela, Spain). Spectra were processed with 7 Hz line broadening. The orthophosphate peak was standardized to 6.00 ppm. Specific ^31^P peaks were identified through spiking experiments^103,104^. To locate their chemical shifts, 0.60 mL of 5 g/L phytic acid, α-glycerophosphate, β-glycerophosphate, choline phosphate, and adenosine 5′ monophosphate, were added separately to the dissolved soil extracts and analyzed under the same experimental conditions.

Orthophosphate diester degradation products (i.e., α-glycerophosphate and β-glycerophosphate) were removed from the monoester group and included with diesters^69,72,100^. The rate of total P recovery in the NaOH-EDTA extracted NMR samples was estimated by digesting the extracts with 5.5 M H_2_SO_4_ and K_2_S_2_O_8_. The P concentrations were measured using a modified molybdate blue method^86^ and divided by total P to calculate percent recovery.

### Statistical and kinetic analyses

Fisher’s Least Significant Difference (LSD) tests were conducted on acid and alkaline phosphatase activity, labile inorganic P, and percent organic P within each sampling period. The purpose of this analysis was to determine whether these parameters were significantly different, as a function of treatment and amendment, at a significance level of 0.05. Furthermore, linear fits were conducted for each sampling time between acid/alkaline phosphatase activity and the amount of nitrogen added to the soil to assess the correlations between these two factors and their significance. All statistical analyses were completed on Origin Lab 2019 (Northampton, MA, USA).

After conducting P mineralization and immobilization assays on the kinetic samples, the labile P data was fit to a zero-order kinetic model, P _min_ = k_0_ · t, to estimate the rate of P mineralization, where P_min_ refers to the cumulative P mineralized, or changes in labile inorganic P, k_0_ refers to a constant rate of mineralization, and t refers to time^105^. P_min_ versus time of incubation was plotted, and the model was then fit over the plot using linear regression. This kinetic equation was successfully used to model P mineralization in undried, fertilized soil, like the conditions in this present study^105^, as well as floodplain sediments^61^.

## Supplementary Information

Supplementary Information.

## References

[1] Turner BL, Condron LM (2013). Pedogenesis, nutrient dynamics, and ecosystem development: The legacy of T.W. Walker and J.K. Syers. Plant Soil.

[2] Turner BL (2013). Soil microbial biomass and the fate of phosphorus during long-term ecosystem development. Plant Soil.

[3] Walker TW, Syers JK (1976). The fate of phosphorus during pedogenesis. Geoderma.

[4] Fu D, Wu X, Duan C, Chadwick DR, Jones DL (2020). Response of soil phosphorus fractions and fluxes to different vegetation restoration types in a subtropical mountain ecosystem. CATENA.

[5] Condron, L. M., Turner, B. L. & Cade-Menun, B. J. Chemistry and dynamics of soil organic phosphorus. In *Phosphorus: Agriculture and the environment, Agronomy Monograph no. 46* 87–121 (American Society of Agronomy, Crop Science Society of America, and Soil Science Society of America, 2005) 10.2134/agronmonogr46.c4.

[6] Arenberg MR, Arai Y (2019). Uncertainties in soil physicochemical factors controlling phosphorus mineralization and immobilization processes. Adv. Agron..

[7] Achat DL, Augusto L, Bakker MR, Gallet-Budynek A, Morel C (2012). Microbial processes controlling P availability in forest spodosols as affected by soil depth and soil properties. Soil Biol. Biochem..

[8] Lockaby BG, Murphy AL, Somers GL (1996). Hydroperiod influences on nutrient dynamics in decomposing litter of a floodplain forest. Soil Sci. Soc. Am. J..

[9] Harrison AF (1982). Labile organic phosphorus mineralization in relationship to soil properties. Soil Biol. Biochem..

[10] Mosheim, R. Fertilizer Use and Price. *USDA Economic Research Service* https://www.ers.usda.gov/data-products/fertilizer-use-and-price/ (2019).

[11] Vitousek PM, Porder S, Houlton BZ, Chadwick OA (2010). Terrestrial phosphorus limitation: Mechanisms, implications, and nitrogen-phosphorus interactions. Ecol. Appl..

[12] Margalef O (2017). Global patterns of phosphatase activity in natural soils. Sci. Rep..

[13] Wang YP, Houlton BZ, Field CB (2007). A model of biogeochemical cycles of carbon, nitrogen, and phosphorus including symbiotic nitrogen fixation and phosphatase production. Glob. Biogeochem. Cycles.

[14] Saiya-Cork KR, Sinsabaugh RL, Zak DR (2002). The effects of long term nitrogen deposition on extracellular enzyme activity in an Acer saccharum forest soil. Soil Biol. Biochem..

[15] McGill WB, Cole CV (1981). Comparative aspects of cycling of organic C, N, S and P through soil organic matter. Geoderma.

[16] Olander LP, Vitousek PM (2000). Regulation of soil phosphatase and chitinase activity by N and P availability. Biogeochemistry.

[17] Arenberg MR, Arai Y (2020). Immobilization of agricultural phosphorus in temperate floodplain soils of Illinois, USA. Biogeochemistry.

[18] Symanowicz B, Kalembasa S, Niedbała M, Toczko M, Skwarek K (2018). Fertilisation of pea (*Pisum sativum* L.) with nitrogen and potassium and its effect on soil enzymatic activity. J. Elem..

[19] Chen X (2019). Soil alkaline phosphatase activity and bacterial phoD gene abundance and diversity under long-term nitrogen and manure inputs. Geoderma.

[20] Johnson D, Leake JR, Read DJ (2005). Liming and nitrogen fertilization affects phosphatase activities, microbial biomass and mycorrhizal colonisation in upland grassland. Plant Soil.

[21] DeForest JL, Zak DR, Pregitzer KS, Burton AJ (2004). Atmospheric nitrate deposition, microbial community composition, and enzyme activity in northern hardwood forests. Soil Sci. Soc. Am. J..

[22] Phoenix GK (2004). Simulated pollutant nitrogen deposition increases P demand and enhances root-surface phosphatase activities of three plant functional types in a calcareous grassland. New Phytol..

[23] Turner BL, Baxter R, Whitton BA (2002). Seasonal phosphatase activity in three characteristic soils of the English uplands polluted by long-term atmospheric nitrogen deposition. Environ. Pollut..

[24] Widdig M (2019). Nitrogen and phosphorus additions alter the abundance of phosphorus-solubilizing bacteria and phosphatase activity in grassland soils. Front. Environ. Sci..

[25] Zhang X (2018). Contrasting responses of phosphatase kinetic parameters to nitrogen and phosphorus additions in forest soils. Funct. Ecol..

[26] Siwik-Ziomek A, Lemanowicz J (2016). The influence of fertilization with phosphorus, sulphate, carbon and nitrogen content on hydrolases activities in soil. Polish J. Soil Sci..

[27] Godin AM, Lidher KK, Whiteside MD, Jones MD (2015). Control of soil phosphatase activities at millimeter scales in a mixed paper birch-Douglas-fir forest: The importance of carbon and nitrogen. Soil Biol. Biochem..

[28] Iyyemperumal K, Shi W (2008). Soil enzyme activities in two forage systems following application of different rates of swine lagoon effluent or ammonium nitrate. Appl. Soil Ecol..

[29] Deng Q, Hui D, Dennis S, Reddy KC (2017). Responses of terrestrial ecosystem phosphorus cycling to nitrogen addition: A meta-analysis. Glob. Ecol. Biogeogr..

[30] Wei K, Sun T, Tian J, Chen Z, Chen L (2018). Soil microbial biomass, phosphatase and their relationships with phosphorus turnover under mixed inorganic and organic nitrogen addition in a *Larix gmelinii* plantation. For. Ecol. Manage..

[31] Johnson D, Leake JR, Lee JA (1999). The effects of quantity and duration of simulated pollutant nitrogen deposition on root-surface phosphatase activities in calcareous and acid grasslands: A bioassay approach. New Phytol..

[32] Chen J (2020). Long-term nitrogen loading alleviates phosphorus limitation in terrestrial ecosystems. Glob. Chang. Biol..

[33] Ma SN (2018). High ammonium loading can increase alkaline phosphatase activity and promote sediment phosphorus release: A two-month mesocosm experiment. Water Res..

[34] Marklein AR, Houlton BZ (2012). Nitrogen inputs accelerate phosphorus cycling rates across a wide variety of terrestrial ecosystems. New Phytol..

[35] Xu Z (2020). Carbon addition reduces labile soil phosphorus by increasing microbial biomass phosphorus in intensive agricultural systems. Soil Use Manag..

[36] Ratliff TJ, Fisk MC (2016). Phosphatase activity is related to N availability but not P availability across hardwood forests in the northeastern United States. Soil Biol. Biochem..

[37] Heuck C (2018). Effects of long-term nitrogen addition on phosphorus cycling in organic soil horizons of temperate forests. Biogeochemistry.

[38] Kruse J (2015). Innovative methods in soil phosphorus research: A review. J. Plant Nutr. Soil Sci..

[39] Vincent JB, Crowder MW, Averill BA (1992). Hydrolysis of phosphate monoesters: A biological problem with multiple chemical solutions. Trends Biochem. Sci..

[40] Nannipieri, P., Giagnoni, L., Landi, L. & Renella, G. Role of phosphatase enzymes in soil. In *Phosphorus in Action*, Vol. 26, 215–243 (Soil Biology, 2011).

[41] Spohn M, Kuzyakov Y (2013). Phosphorus mineralization can be driven by microbial need for carbon. Soil Biol. Biochem..

[42] Bünemann EK, Augstburger S, Frossard E (2016). Dominance of either physicochemical or biological phosphorus cycling processes in temperate forest soils of contrasting phosphate availability. Soil Biol. Biochem..

[43] Zhang C (2017). Contrasting effects of ammonium and nitrate additions on the biomass of soil microbial communities and enzyme activities in subtropical China. Biogeosciences.

[44] Shao T, Zhao JJ, Liu A, Long X, Rengel Z (2020). Effects of soil physicochemical properties on microbial communities in different ecological niches in coastal area. Appl. Soil Ecol..

[45] Min K, Kang H, Lee D (2011). Effects of ammonium and nitrate additions on carbon mineralization in wetland soils. Soil Biol. Biochem..

[46] Li FR, Liu LL, Liu JL, Yang K (2019). Abiotic and biotic controls on dynamics of labile phosphorus fractions in calcareous soils under agricultural cultivation. Sci. Total Environ..

[47] Kuypers MMM, Marchant HK, Kartal B (2018). The microbial nitrogen-cycling network. Nat. Rev. Microbiol..

[48] Hachiya T, Sakakibara H (2017). Interactions between nitrate and ammonium in their uptake, allocation, assimilation, and signaling in plants. J. Exp. Bot..

[49] Kirchman DL, Ducklow HW, McCarthy JJ, Garside C (1994). Biomass and nitrogen uptake by heterotrophic bacteria during the spring phytoplankton bloom in the North Atlantic Ocean. Deep. Res. Part.

[50] Mallarino AP (1997). Interpretation of soil phosphorus tests for corn in soils with varying pH and calcium carbonate content. J. Prod. Agric..

[51] Dossa EL (2009). Carbon, nitrogen and phosphorus mineralization potential of semiarid Sahelian soils amended with native shrub residues. Geoderma.

[52] Fabre A, Pinay G, Ruffinoni C (1996). Seasonal changes in inorganic and organic phosphorus in the soil of a riparian forest. Biogeochemistry.

[53] Fiorentino I (2003). Initial responses of phosphorus biogeochemistry to calcium addition in a northern hardwood forest ecosystem. Can. J. For. Res..

[54] Chauhan BS, Stewart JWB, Paul EA (1979). Effect of carbon additions on soil labile inorganic, organic, and microbially held phosphate. Can. J. Soil Sci..

[55] White RE, Ayoub AT (1983). Decomposition of plant residues of variable C/P ratio and the effect on soil phosphate availability. Plant Soil.

[56] Iyamuremye F (2000). Carbon, nitrogen and phosphorus mineralization potential of native agroforestry plant residues in soils of Senegal. Arid Soil Res. Rehabil..

[57] Sharpley AN, Smith SJ (1989). Prediction of bioavailable phosphorus loss in agricultural runoff. J. Environ. Qual..

[58] Van Meeteren MJM, Tietema A, Westerveld JW (2007). Regulation of microbial carbon, nitrogen, and phosphorus transformations by temperature and moisture during decomposition of *Calluna vulgaris* litter. Biol. Fertil. Soils.

[59] Saggar S, Parfitt RL, Salt G, Skinner MF (1998). Carbon and phosphorus transformations during decomposition of pine forest floor with different phosphorus status. Biol. Fertil. Soils.

[60] Bünemann EK, Marschner P, McNeill AM, McLaughlin MJ (2007). Measuring rates of gross and net mineralisation of organic phosphorus in soils. Soil Biol. Biochem..

[61] Bai J (2019). In-situ organic phosphorus mineralization in sediments in coastal wetlands with different flooding periods in the Yellow River Delta, China. Sci. Total Environ..

[62] Jianbo S (2011). Phosphorus dynamics: From soil to plant. Plant Physiol..

[63] van Breemen N, Mulder J, Driscoll CT (1983). Acidification and alkalinization of soils. Plant Soil.

[64] Noe GB, Hupp CR, Rybicki NB (2013). Hydrogeomorphology influences soil nitrogen and phosphorus mineralization in floodplain wetlands. Ecosystems.

[65] Reitzel K (2012). Diagenesis of settling seston: Identity and transformations of organic phosphorus. J. Environ. Monit..

[66] Hinedi ZR, Chang AC, Lee RWK (1988). Mineralization of phosphorus in sludge-amended soils monitored by phosphorus-31-nuclear magnetic resonance spectroscopy. Soil Sci. Soc. Am. J..

[67] Tate KR, Tinsley J, Darbyshire JF (1984). The biological transformation of P in soil. Biological Processes and Soil Fertility.

[68] McDowell RW, Condron LM, Stewart I, Cave V (2005). Chemical nature and diversity of phosphorus in New Zealand pasture soils using ^31^P nuclear magnetic resonance spectroscopy and sequential fractionation. Nutr. Cycl. Agroecosyst..

[69] Abdi D (2019). A 31 P-NMR spectroscopic study of phosphorus forms in two phosphorus-fertilized grassland soils in eastern Canada. Can. J. Soil Sci..

[70] Cade-Menun BJ (2015). Improved peak identification in ^31^P-NMR spectra of environmental samples with a standardized method and peak library. Geoderma.

[71] Stutter MI (2015). Land use and soil factors affecting accumulation of phosphorus species in temperate soils. Geoderma.

[72] Schneider KD, Cade-Menun BJ, Lynch DH, Voroney RP (2016). Soil phosphorus forms from organic and conventional forage fields. Soil Sci. Soc. Am. J..

[73] Cheesman AW, Turner BL, Inglett PW, Reddy KR (2010). Phosphorus transformations during decomposition of wetland macrophytes. Environ. Sci. Technol..

[74] Turner BL, Paphazy MJ, Haygarth PM, Mckelvie ID (2002). Inositol phosphates in the environment. Philos. Trans. R. Soc. Biol. Sci..

[75] Giles C, Cade-Menun B, Hill J (2011). The inositol phosphates in soils and manures: Abundance, cycling, and measurement. Can. J. Soil Sci..

[76] Currey PM (2010). Turnover of labile and recalcitrant soil carbon differ in response to nitrate and ammonium deposition in an ombrotrophic peatland. Glob. Chang. Biol..

[77] Bloom AJ, Burger M, Kimball BA, Pinter PJ (2014). Nitrate assimilation is inhibited by elevated carbon dioxide in field-grown wheat. Nat. Clim. Chang..

[78] Polit JI (1992). Structure and Composition of Woody Debris in a Central Illinois Floodplain Forest.

[79] Tsai J, David MB, Darmody RG (2014). Twenty-three-year changes in upland and bottomland forest soils of central Illinois. Soil Sci..

[80] Thomas GW, Sparks DL (1996). Soil pH and soil acidity. Methods of Soil Analysis, Part 3, Chemical Analysis.

[81] Suarez DL, Sparks DL (1996). Beryllium, magnesium, calcium, strontium, and barium. Methods of Soil Analysis Part 3: Chemical Methods.

[82] Ross, D. J. Recommended soil tests for determining exchange capacity. In *Recommended Soil Testing Procedures for the Northeastern United States. Northeastern Regional Bulletin #493.* (eds Sims, J. T. & Wolf, A.) 62–69 (1995).

[83] Schulte EE, Hopkins BG, Magdoff FR, Tabatabai MA, Hanlon E (1996). Estimation of soil organic matter by weight Loss-On Ignition. Soil Organic Matter: Analysis and Interpretation.

[84] Allison LE (1960). Wet-combustion apparatus and procedure for organic and inorganic carbon in soil. Soil Sci. Soc. Am. J..

[85] Bowman RA (1989). A sequential extraction procedure with concentrated sulfuric acid and dilute base for soil organic phosphorus. Soil Sci. Soc. Am. J..

[86] He ZL, Baligar VC, Ritchey KD, Martens DC (1998). Determination of soluble phosphorus in the presence of organic ligands or fluoride. Soil Sci. Soc. Am. J..

[87] Miller AP, Arai Y (2016). Comparative evaluation of phosphate spectrophotometric methods in soil test phosphorus extracting solutions. Soil Sci. Soc. Am. J..

[88] Tabatabai MA, Bremner JM (1969). Use of p-nitrophenyl phosphate for assay of soil phosphatase activity. Soil Biol. Biochem..

[89] Olsen SR, Sommers LE, Page AL, Miller RH, Keeney DR (1982). Phosphorus. Methods of Soil Analysis. Part 2: Chemical and Microbiological Properties.

[90] Cade-Menun B, Liu CW (2014). Solution phosphorus-31 nuclear magnetic resonance spectroscopy of soils from 2005 to 2013: A review of sample preparation and experimental parameters. Soil Sci. Soc. Am. J..

[91] Cade-Menun BJ, Liu CW, Nunlist R, McColl JG (2002). Soil and litter phosphorus-31 nuclear magnetic resonance spectroscopy: Extractants, metals, and phosphorus relaxation times. J. Environ. Qual..

[92] Zheng ZM, Zhang TQ, Whalen J (2012). Soil phosphorus tests and transformation analysis to quantify plant availability: a review. Soil Fertility Improvement and Integrated Nutrient Management—A Global Perspective.

[93] Sims JT, Pierzynski GM (2000). Soil test phosphorus: Olsen P. Methods of Phosphorus Analysis for Soils, Sediments, Residuals, and Waters.

[94] Dakora FD, Phillips DA (2002). Root exudates as mediators of mineral acquisition in low-nutrient environments. Plant Soil.

[95] Rosling A (2016). Phosphorus cycling in deciduous forest soil differs between stands dominated by ecto-and arbuscular mycorrhizal trees. New Phytol..

[96] George TS (2018). Organic phosphorus in the terrestrial environment: A perspective on the state of the art and future priorities. Plant Soil.

[97] Tarafdar JC, Claassen N (1988). Organic phosphorus compounds as a phosphorus source for higher plants through the activity of phosphatases produced by plant roots and microorganisms. Biol. Fertil. Soils.

[98] Mulvaney, R. L. Nitrogen—inorganic forms. In *Methods of Soil Analysis. Part 3. Chemical Method* 1123–1184 (Soil Science Society of America and American Society of Agronomy, 1996) 10.1201/9780203500477-48.

[99] Patey MD (2008). Determination of nitrate and phosphate in seawater at nanomolar concentrations. TrAC Trends Anal. Chem..

[100] Jiang X (2017). Colloid-bound and dissolved phosphorus species in topsoil water extracts along a grassland transect from Cambisol to Stagnosol. Biogeosciences.

[101] Noack SR, McLaughlin MJ, Smernik RJ, McBeath TM, Armstrong RD (2012). Crop residue phosphorus: Speciation and potential bio-availability. Plant Soil.

[102] Noack SR, Smernik RJ, McBeath TM, Armstrong RD, McLaughlin MJ (2014). Assessing crop residue phosphorus speciation using chemical fractionation and solution ^31^P nuclear magnetic resonance spectroscopy. Talanta.

[103] Doolette AL, Smernik RJ, Dougherty WJ (2009). Spiking improved solution phosphorus-31 nuclear magnetic resonance identification of soil phosphorus compounds. Soil Sci. Soc. Am. J..

[104] McDowell RW, Cade-Menun B, Stewart I (2007). Organic phosphorus speciation and pedogenesis: Analysis by solution ^31^P nuclear magnetic resonance spectroscopy. Eur. J. Soil Sci..

[105] Grierson PF, Comerford NB, Jokela EJ (1998). Phosphorus mineralization kinetics and response of microbial phosphorus to drying and rewetting in a Florida spodosol. Soil Biol. Biochem..

