# Supplementary material for: Nitrogen species specific phosphorus mineralization in temperate floodplain soils
Source: Sci Rep. 2021 Aug 31;11:17430. doi: 10.1038/s41598-021-96885-5 (PMC8408222; doi:10.1038/s41598-021-96885-5)
Supplement: Supplementary file 1 — Supplementary Information. [file 41598_2021_96885_MOESM1_ESM.docx]

**Supplemental Materials**

**Nitrogen Species Specific Phosphorus Mineralization in Temperate Floodplain Soils**

Mary R. Arenberg^†^, and Yuji Arai*^, †^

^†^ Department of Natural Resources and Environmental Sciences, University of Illinois at Urbana-Champaign, Urbana, IL 61801, USA

**Table of content**

**Table A1.** Results of Fisher’s Least Significant Difference (LSD) Test of acid phosphatase of N treatment. The data in Figure 1 were used for the statistical analysis. P values of Fisher’s LSD tests (P≤0.05) were reported. Numbers in bold and italics indicate a statistical difference between the two treatments at the same sampling period.

**Table A2.** Results of Fisher’s Least Significant Difference (LSD) Test of acid phosphatase of N amendment rate. The data in Figure 1 were used for the statistical analysis. P values of Fisher’s LSD tests (P≤0.05) were reported. Numbers in bold and italics indicate a statistical difference between the two treatments at the same sampling period.

**Table A3.** Linear fitting between acid phosphatase activity (µmol pNP g^-1^ hr^-1^) and nitrogen added (mg N kg^-1^) was conducted. The trendlines and corresponding coefficients of determination (R^2^) are presented below. R^2^ values in bold and italics indicate that the slope is significantly different from zero at the 0.05 level.

**Table A4.** Results of Fisher’s Least Significant Difference (LSD) Test of alkaline phosphatase of N treatment. The data in Figure 1 were used for the statistical analysis. P values of Fisher’s LSD tests (P≤0.05) were reported. Numbers in bold and italics indicate a statistical difference between the two treatments at the same sampling period.

**Table A5.** Results of Fisher’s Least Significant Difference (LSD) Test of alkaline phosphatase of N amendment rate. The data in Figure 1 were used for the statistical analysis. P values of Fisher’s LSD tests (P≤0.05) were reported. Numbers in bold and italics indicate a statistical difference between the two treatments at the same sampling period.

**Table A6.** Linear fitting between alkaline phosphatase activity (µmol pNP g^-1^ hr^-1^) and nitrogen added (mg N kg^-1^) was conducted. The trendlines and corresponding coefficients of determination (R^2^) are presented below. R^2^ values in bold and italics indicate that the slope is significantly different from zero at the 0.05 level.

**Table A7.** Results of Fisher’s Least Significant Difference (LSD) Test of labile inorganic P of N treatment. The data in Figure 2 were used for the statistical analysis. P values of Fisher’s LSD tests (P≤0.05) were reported. Numbers in bold and italics indicate a statistical difference between the two treatments at the same sampling period.

**Table A8.** Results of Fisher’s Least Significant Difference (LSD) Test of labile inorganic P of N amendment rate. The data in Figure 2 were used for the statistical analysis. P values of Fisher’s LSD tests (P≤0.05) were reported. Numbers in bold and italics indicate a statistical difference between the two treatments at the same sampling period.

**Table A9.** Results of Fisher’s Least Significant Difference (LSD) Test of percent labile P of N treatment. The data in Figure 3-B were used for the statistical analysis. P values of Fisher’s LSD tests (P≤0.05) were reported. Numbers in bold and italics indicate a statistical difference between the two treatments at the same sampling period.

**Table A10.** 10. identified in the solution ^31^P NMR spectra in this study.

**Figure A1.** Solution ^31^P NMR spectra of NaOH-EDTA extracted soil: A) the control on day 0, spiked with B) phytate, C) α-glycerophosphate (i.e., DL-α-Glycerol phosphate magnesium salt hydrate, 85%), D) β-glycerophosphate, E) choline phosphate, and F) adenosine 5’ monophosphate.

**Figure A2.** A) ammonium and B) nitrate on days 0, 20, and 40 of the incubation as a function of ammonium amendment rate: 1.5 mg kg^-1^, 2.5 mg kg^-1^, and 3.5 mg kg^-1^. All analyses were conducted in duplicate. The error bars indicate the standard deviation.

**Table A1.** Results of Fisher’s Least Significant Difference (LSD) Test of acid phosphatase of N treatment. The data in Figure 1 were used for the statistical analysis. P values of Fisher’s LSD tests (P≤0.05) were reported. Numbers in bold and italics indicate a statistical difference between the two treatments at the same sampling period.

| **N Treatment** | **Incubation time (days)** | | | | | | | | | | | | | |
| --- | --- | --- | --- | --- | --- | --- | --- | --- | --- | --- | --- | --- | --- | --- |
|  | **2** | | **5** | | **10** | | **15** | | **20** | | **30** | | **40** | |
|  | **Control** | **NO_3_-N** | **Control** | **NO_3_-N** | **Control** | **NO_3_-N** | **Control** | **NO_3_-N** | **Control** | **NO_3_-N** | **Control** | **NO_3_-N** | **Control** | **NO_3_-N** |
| **Control** | --- | --- | --- | --- | --- | --- | --- | --- | --- | --- | --- | --- | --- | --- |
| **NO_3_-N** | 0.210 | ---- | 0.371 | ---- | 0.538 | ---- | 0.171 | ---- | 0.277 | ---- | 0.230 | ---- | 0.476 | ---- |
| **NH_4_-N** | 0.195 | ***0.004*** | ***0.021*** | ***0.029*** | ***0.030*** | ***0.023*** | 0.725 | 0.147 | ***0.037*** | 0.100 | ***0.003*** | ***0.005*** | 0.219 | 0.432 |

**Table A2.** Results of Fisher’s Least Significant Difference (LSD) Test of acid phosphatase of N amendment rate. The data in Figure 1 were used for the statistical analysis. P values of Fisher’s LSD tests (P≤0.05) were reported. Numbers in bold and italics indicate a statistical difference between the two treatments at the same sampling period.

| **Amendment Rate**  **(mg N kg^-1^)** | **Incubation time (days)** | | | | | | | | | | | | | | | | | | | | | |
| --- | --- | --- | --- | --- | --- | --- | --- | --- | --- | --- | --- | --- | --- | --- | --- | --- | --- | --- | --- | --- | --- | --- |
|  | **2** | | | **5** | | | **10** | | | **15** | | | **20** | | | **30** | | | | **40** | | |
|  | **0** | **1.5** | **2.5** | **0** | **1.5** | **2.5** | **0** | **1.5** | **2.5** | **0** | **1.5** | **2.5** | **0** | **1.5** | **2.5** | **0** | **1.5** | **2.5** | **0** | | **1.5** | **2.5** |
| **0** | --- | --- | --- | --- | --- | --- | --- | --- | --- | --- | --- | --- | --- | --- | --- | --- | --- | --- | --- | | --- | --- |
| **1.5** | 0.738 | ---- | ---- | ***0.047*** | ---- | ---- | 0.317 | ---- | ---- | 0.056 | ---- | ---- | 0.091 | ---- | ---- | 0.051 | ---- | ---- | 0.539 | | ---- | ---- |
| **2.5** | 0.481 | 0.219 | ---- | 0.217 | 0.266 | ---- | 0.150 | 0.545 | ---- | 0.972 | ***0.023*** | ---- | 0.171 | 0.631 | ---- | ***0.024*** | 0.592 | ---- | 0.270 | | 0.527 | ---- |
| **3.5** | 0.655 | 0.888 | 0.177 | 0.135 | 0.446 | 0.706 | 0.088 | 0.322 | 0.685 | 0.659 | 0.062 | 0.562 | 0.118 | 0.846 | 0.773 | ***0.037*** | 0.822 | 0.753 | 0.295 | | 0.578 | 0.939 |

**Table A3.** Linear fitting between acid phosphatase activity (µmol pNP g^-1^ hr^-1^) and nitrogen added (mg N kg^-1^) was conducted. The trendlines and corresponding coefficients of determination (R^2^) are presented below. R^2^ values in bold and italics indicate that the slope is significantly different from zero at the 0.05 level.

| **Nitrogen Treatment** | **Time (Days)** | **Acid Phosphatase Activity (****µmol pNP g^-1^ hr^-1^) vs. Nitrogen Added (mg N kg^-1^) Trendline** | **R^2^** |
| --- | --- | --- | --- |
| Nitrate | 2 | y = -0.237x + 5.99 | 0.741 |
|  | 5 | y = -0.056x + 4.98 | 0.530 |
|  | 10 | y = 0.041+ 4.42 | 0.530 |
|  | 15 | y = -0.140x + 5.90 | 0.178 |
|  | 20 | y = 0.068x + 5.06 | 0.299 |
|  | 30 | y = 0.107x + 4.74 | ***0.968*** |
|  | 40 | y = -0.040x + 5.07 | 0.056 |
| Ammonium | 2 | y = 0.219x + 5.93 | 0.777 |
|  | 5 | y = 0.401x + 4.77 | ***0.984*** |
|  | 10 | y = 0.342x + 4.37 | ***0.985*** |
|  | 15 | y = 0.160x + 5.47 | 0.353 |
|  | 20 | y = 0.264x + 5.05 | ***0.952*** |
|  | 30 | y = 0.206x + 4.97 | 0.511 |
|  | 40 | y = 0.142x + 4.93 | 0.551 |

**Table A4.** Results of Fisher’s Least Significant Difference (LSD) Test of alkaline phosphatase of N treatment. The data in Figure 1 were used for the statistical analysis. P values of Fisher’s LSD tests (P≤0.05) were reported. Numbers in bold and italics indicate a statistical difference between the two treatments at the same sampling period.

| **N Treatment** | **Time (days)** | | | | | | | | | | | | | |
| --- | --- | --- | --- | --- | --- | --- | --- | --- | --- | --- | --- | --- | --- | --- |
|  | **2** | | **5** | | **10** | | **15** | | **20** | | **30** | | **40** | |
|  | **Control** | **NO_3_-N** | **Control** | **NO_3_-N** | **Control** | **NO_3_-N** | **Control** | **NO_3_-N** | **Control** | **NO_3_-N** | **Control** | **NO_3_-N** | **Control** | **NO_3_-N** |
| **Control** | --- | --- | --- | --- | --- | --- | --- | --- | --- | --- | --- | --- | --- | --- |
| **NO_3_-N** | 0.518 | ---- | 0.615 | ---- | 0.974 | ---- | 0.161 | ---- | 0.643 | ---- | 0.678 | ---- | 0.412 | ---- |
| **NH_4_-N** | 0.272 | 0.500 | 0.119 | 0.123 | 0.169 | 0.059 | ***0.037*** | 0.228 | ***0.005*** | ***0.001*** | 0.692 | 0.979 | 0.740 | 0.482 |

**Table A5.** Results of Fisher’s Least Significant Difference (LSD) Test of alkaline phosphatase of N amendment rate. The data in Figure 1 were used for the statistical analysis. P values of Fisher’s LSD tests (P≤0.05) were reported. Numbers in bold and italics indicate a statistical difference between the two treatments at the same sampling period.

| **Amendment Rate**  **(mg N kg^-1^)** | **Time (days)** | | | | | | | | | | | | | | | | | | | | | |
| --- | --- | --- | --- | --- | --- | --- | --- | --- | --- | --- | --- | --- | --- | --- | --- | --- | --- | --- | --- | --- | --- | --- |
|  | **2** | | | **5** | | | **10** | | | **15** | | | **20** | | | **30** | | | **40** | | |  |
|  | **0** | **1.5** | **2.5** | **0** | **1.5** | **2.5** | **0** | **1.5** | **2.5** | **0** | **1.5** | **2.5** | **0** | **1.5** | **2.5** | **0** | **1.5** | **2.5** | **0** | **1.5** | **2.5** | |
| **0** | ---- | ---- | ---- | ---- | ---- | ---- | ---- | ---- | ---- | ---- | ---- | ---- | ---- | ---- | ---- | ---- | ---- | ---- | ---- | ---- | ---- | |
| **1.5** | 0.176 | ---- | ---- | 0.132 | ---- | ---- | 0.816 | ---- | ---- | 0.081 | ---- | ---- | 0.149 | ---- | ---- | 0.938 | ---- | ---- | 0.828 | ---- | ---- | |
| **2.5** | 0.934 | 0.124 | ---- | 0.282 | 0.544 | ---- | 0.355 | 0.392 | ---- | 0.055 | 0.777 | ---- | 0.070 | 0.572 | ---- | 0.686 | 0.558 | ---- | 0.602 | 0.706 | ---- | |
| **3.5** | 0.320 | 0.621 | 0.267 | 0.720 | 0.149 | 0.368 | 0.415 | 0.471 | 0.887 | 0.181 | 0.546 | 0.382 | ***0.043*** | 0.365 | 0.722 | 0.419 | 0.286 | 0.612 | 0.374 | 0.405 | 0.640 | |

**Table A6.** Linear fitting between alkaline phosphatase activity (µmol pNP g^-1^ hr^-1^) and nitrogen added (mg N kg^-1^) was conducted. The trendlines and corresponding coefficients of determination (R^2^) are presented below. R^2^ values in bold and italics indicate that the slope is significantly different from zero at the 0.05 level.

| **Treatment** | **Day** | **Alkaline Phosphatase Activity (µmol pNP g^-1^ hr^-1^) vs. Nitrogen Added (mg N kg^-1^) Trendline** | **R^2^** |
| --- | --- | --- | --- |
| Nitrate | 2 | y = 0.072x + 6.86 | 0.047 |
|  | 5 | y = -0.014x + 8.30 | 0.002 |
|  | 10 | y = -0.110x + 7.51 | 0.294 |
|  | 15 | y = 0.050x + 8.04 | 0.010 |
|  | 20 | y = -0.276x + 7.94 | 0.645 |
|  | 30 | y = 0.040x + 7.48 | 0.210 |
|  | 40 | y = -0.008x + 7.49 | 0.002 |
| Ammonium | 2 | y = -0.046x + 7.34 | 0.017 |
|  | 5 | y = 0.235x + 8.02 | ***0.976*** |
|  | 10 | y = 0.350x + 7.03 | 0.694 |
|  | 15 | y = 0.403x + 7.44 | ***0.968*** |
|  | 20 | y = 0.527x + 6.80 | ***0.920*** |
|  | 30 | y = 0.080x + 7.46 | 0.410 |
|  | 40 | y = 0.096x + 7.05 | 0.236 |

**Table A7.** Results of Fisher’s Least Significant Difference (LSD) Test of labile inorganic P of N treatment. The data in Figure 2 were used for the statistical analysis. P values of Fisher’s LSD tests (P≤0.05) were reported. Numbers in bold and italics indicate a statistical difference between the two treatments at the same sampling period.

| **N Treatment** | **Time (days)** | | | | | | | | | | | |
| --- | --- | --- | --- | --- | --- | --- | --- | --- | --- | --- | --- | --- |
|  | **5** | | **10** | | **15** | | **20** | | **30** | | **40** | |
|  | **Control** | **NO_3_-N** | **Control** | **NO_3_-N** | **Control** | **NO_3_-N** | **Control** | **NO_3_-N** | **Control** | **NO_3_-N** | **Control** | **NO_3_-N** |
| **Control** | --- | --- | --- | --- | --- | --- | --- | --- | --- | --- | --- | --- |
| **NO_3_-N** | 0.640 | ---- | 0.373 | ---- | 0.842 | ---- | 0.508 | ---- | 0.474 | ---- | 0.559 | ---- |
| **NH_4_-N** | 0.055 | ***0.004*** | ***0.004*** | ***0.002*** | 0.140 | 0.08 | 0.712 | 0.164 | 0.138 | 0.244 | 0.189 | ***0.019*** |

**Table A8.** Results of Fisher’s Least Significant Difference (LSD) Test of labile inorganic P of N amendment rate. The data in Figure 2 were used for the statistical analysis. P values of Fisher’s LSD tests (P≤0.05) were reported. Numbers in bold and italics indicate a statistical difference between the two treatments at the same sampling period.

| **Amendment Rate**  **(mg N kg^-1^)** | **Time (days)** | | | | | | | | | | | | | | | | | |
| --- | --- | --- | --- | --- | --- | --- | --- | --- | --- | --- | --- | --- | --- | --- | --- | --- | --- | --- |
|  | **5** | | | **10** | | | **15** | | | **20** | | | **30** | | | **40** | | |
|  | **0** | **1.5** | **2.5** | **0** | **1.5** | **2.5** | **0** | **1.5** | **2.5** | **0** | **1.5** | **2.5** | **0** | **1.5** | **2.5** | **0** | **1.5** | **2.5** |
| **0** | --- | --- | --- | --- | --- | --- | --- | --- | --- | --- | --- | --- | --- | --- | --- | --- | --- | --- |
| **1.5** | 0.305 | ---- | ---- | 0.167 | ---- | ---- | 0.543 | ---- | ---- | 1 | ---- | ---- | 0.352 | ---- | ---- | 0.678 | ---- | ---- |
| **2.5** | 0.211 | ***0.015*** | ---- | ***0.025*** | 0.182 | ---- | 0.605 | 0.909 | ---- | 0.511 | 0.423 | ---- | 0.131 | 0.426 | ---- | 0.403 | 0.596 | ---- |
| **3.5** | 0.058 | ***0.003*** | 0.338 | ***0.028*** | 0.207 | 0.932 | .199 | 0.371 | 0.320 | 0.809 | 0.767 | 0.282 | 0.491 | 0.753 | 0.277 | 0.880 | 0.492 | 0.237 |

**Table A9.** Results of Fisher’s Least Significant Difference (LSD) Test of percent organic P of N treatment. The data in Figure 3B were used for the statistical analysis. P values of Fisher’s LSD tests (P≤0.05) were reported. Numbers in bold and italics indicate a statistical difference between the two treatments at the same sampling period.

| **N Treatment** | **Time (days)** | | | | | |
| --- | --- | --- | --- | --- | --- | --- |
|  | **10** | | **20** | | **40** | |
|  | **Control** | **NO_3_-N** | **Control** | **NO_3_-N** | **Control** | **NO_3_-N** |
| **Control** | --- | --- | --- | --- | --- | --- |
| **NO_3_-N** | 0.323 | ---- | 0.123 | ---- | 0.142 | ---- |
| **NH_4_-N** | ***0.017*** | 0.069 | 0.253 | ***0.017*** | ***0.038*** | 0.326 |

**Table A10.** Chemical shifts of peaks of reference P compounds identified in the solution ^31^P NMR spectra in this study.

| **P Species or Compound Class** | **Chemical Shift (ppm)** |
| --- | --- |
| Inorganic P | |
| Orthophosphate | 6.00 |
| Pyrophosphate | -4.10 ± 0.03 |
| Orthophosphate Monoesters | |
| Neo-IHP | 6.41 ± 0.03 |
| Adenosine-5 Phosphate | 4.52 ± 0.02 |
| Choline Phosphate | 3.99 ± 0.01 |
| Scyllo-IHP | 3.75 ± 0.02 |
| Monoester 1 | 5.53 ± 0.03 |
| Monoester 2 | 5.36 ± 0.02 |
| Monoester 3 | 5.15 ± 0.04 |
| Monoester 4 | 4.91 ± 0.03 |
| Monoester 5 | 4.74 ± 0.02 |
| Monoester 6 | 4.30 ± 0.01 |
| Monoester 7 | 4.21 ± 0.02 |
| Monoester 8 | 4.14 ± 0.02 |
| Monoester 9 | 4.08 ± 0.01 |
| Diester Degradation Products | |
| α-Glycerophosphate | 5.04 ± 0.06 |
| β-Glycerophosphate | 4.62 ± 0.04 |
|  |  |
| Diesters | |
| DNA | -0.71 ± 0.04 |


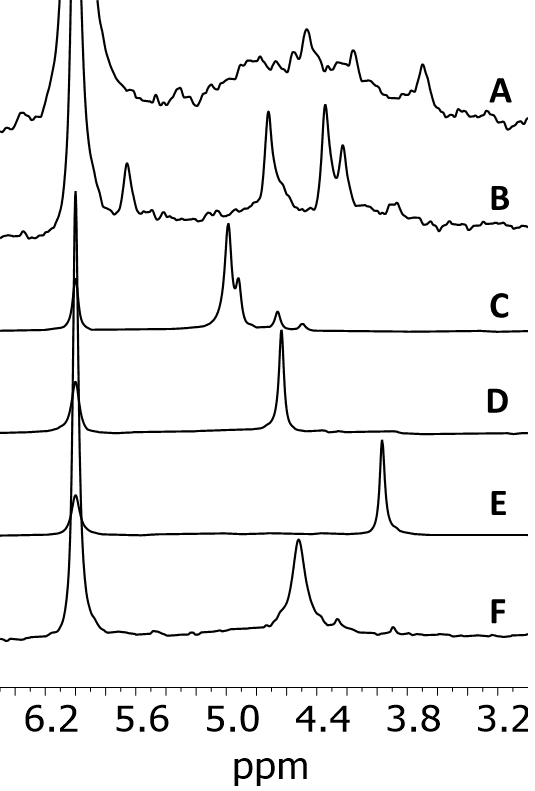


**Figure A1.** Solution ^31^P NMR spectra of NaOH-EDTA extracted soil samples: A) the control on day 0, spiked with B) phytate, C) α-glycerophosphate (i.e., DL-α-Glycerol phosphate magnesium salt hydrate, 85%), D) β-glycerophosphate, E) choline phosphate, and F) adenosine 5’ monophosphate.

**Figure A2.** A) ammonium and B) nitrate on days 0, 20, and 40 of the incubation as a function of ammonium amendment rate: 1.5 mg kg^-1^, 2.5 mg kg^-1^, and 3.5 mg kg^-1^. All analyses were conducted in duplicate. The error bars indicate the standard deviation.
